# Regulation of HSP27 on NF-κB pathway activation may be involved in metastatic hepatocellular carcinoma cells apoptosis

**DOI:** 10.1186/1471-2407-9-100

**Published:** 2009-03-31

**Authors:** Kun Guo, Nan Xiao Kang, Yan Li, Lu Sun, Lin Gan, Feng Jie Cui, Mei Dong Gao, Kun Yin Liu

**Affiliations:** 1 Liver Cancer Institute, Zhongshan Hospital, Fudan University, Shanghai 200032, PR China; 2 Research Center for Cancer, Institute of Biomedical Science, Fudan University, Shanghai 200032, PR China

## Abstract

**Background:**

During the process of metastasis, cells are subjected to various apoptotic stimuli. Aberrant expression of apoptotic regulators often contribute to cell metastasis. Heat shock protein 27(HSP27) is confirmed as an apoptosis regulator, but its antiapoptotic mechanism in metastatic hepatocellular carcinoma (HCC) cells remains unclear.

**Methods:**

Levels of HSP27 protein and its phosphorylation in Hep3B, MHCC97L to MHCC97H cells with different metastatic potentials were determined by western blot analysis. MHCC97H cells were transfected with specific small interference RNA (siRNA) against HSP27. The *in vitro *migration and invasion potentials of cells were evaluated by Transwell assay. The apoptosis ratio of MHCC97H cells was analyzed by TUNEL staining and Flow Cytometry. Alteration of signal transduction pathway after HSP27 knockdown in MHCC97H cells was evaluated through a Human Q Series Signal Transduction in Cancer Gene Array analysis. Nuclear NF-κB contentration and endogenous IKK activity were demonstrated by ELISA assay. The association of IKKα, IKKβ, IκBα with HSP27 and the association between IKKβ and IKKα in MHCC97H cells were determined by co-immunoprecipitation assay followed by western blot analysis.

**Results:**

HSP27 protein and its phosphorylation increased in parallel with enhanced metastatic potentials of HCC cells. siRNA-mediated HSP27 knockdown in MHCC97H significantly suppressed cells migration and invasion *in vitro *and induced cell apoptosis; the prominently altered signal transduction pathway was NF-κB pathway after HSP27 knockdown in MHCC97H cells. Furthermore, inhibition of HSP27 expression led to a significant decrease of nuclear NF-κB contentration and endogenous IKK activity. In addition, HSP27 was associated with IKKα, IKKβ, IκBα in three HCC cells above. ELISA assay and western blot analysis also showed a decrease of the association between IKKβ and IKKα, the association between phosphor-HSP27 and IKK complex, and an increase of total IκBα but reducing tendency of phosphor-IκBα when HSP27 expression was efficiently knocked down in MHCC97H cells.

**Conclusion:**

Altogether, these findings revealed a possible effect of HSP27 on apoptosis in metastatic HCC cells, in which HSP27 may regulate NF-kB pathway activation.

## Background

Metastasis is a complex process that requires the sequential completion of multiple steps in which various molecules are involved [1]. During metastasis process, cells are subjected to various apoptotic stimuli. Thus, in addition to genetic changes that promote up-regulated proliferation, successful metastatic cells must have a decreased sensitivity to apoptotic stimuli [2]. As many metastatic cancer cells exhibit aberrations in levels and functions of key apoptotic regulators, exploiting these alterations to induce tumor cell apoptosis offers a promising therapeutic target [3]. Numerous studies show that NF-κB has anti-apoptotic effects which have been implicated in a variety of biological processes [4]. The association of NF-κB pathway activation with inflammation-associated/driven tumor promotion, progression, and metastasis was demonstrated in several mouse models [5]. It was found that suppressing chronic NF-κB activation at later stages resulted in the apoptotic death of transformed hepatocytes and failure to progress to hepatocellular carcinoma [6].

Heat shock protein 27 (HSP27) is an ATP-independent chaperone that confers protection against apoptosis through various mechanisms, including interacting with and inhibiting components of both stress and death-receptor induced apoptotic pathways [7]. For example, when overexpressed in response to various stimuli, HSP27 facilitates phosphorylated IκBα proteasome mediated proteolysis and enhances NF-κB activity, which could account for its antiapoptotic properties [8]. Moreover, increased levels of HSP27 expression may render tumours more resistant to host defence mechanisms and increase the metastatic potential of tumors cells [9]. Recently, our previous study showed the important effect of HSP27 on metastatic hepatocellular carcinoma (HCC) cells motility [10]. These documents above suggest there may be an internal mechanism which answers for regulation of HSP27 on apoptosis in metastatic HCC cells.

In order to elucidate the assumption, in this study, we demonstrated significant effects of HSP27 on HCC cells motility and apoptosis *in vitro*. Analysis of the cDNA microarray data revealed such effects may be related to the regulation of HSP27 on NF-κB pathway activation in metastatic HCC cells, which was confirmed in further biochemical investigation.

## Methods

### Cell culture

Hep3B cells were maintained in RPMI1640 medium (Gibco-BRL, NY, USA) supplemented with 10% fetal bovine serum (FBS). The other two HCC cell lines with higher (MHCC97-H) and high (MHCC97-L) metastatic potential were established in our institute [11]. Both cells were cultured in high Glucose Dulbecco's modified Eagle's medium (Gibco-BRL, NY, USA) supplemented with 10% fetal calf serum at 37°C in a humidified atmosphere of 95% air and 5% CO_2_.

### RNA interference

HSP27 siRNA duplex (sense:5'-ACGGUCAAGACCAAGGAUGdTdT-3'; antisense: 5'-CAUCCUUGGUCUUGACCGUdTdT-3') were purchased from Cell Signaling Technology. 2 × 10^6 ^cells were transfected with a final concentration (100 nM) of HSP27 siRNA using Lipofectamine 2000 for 24 hr to detect mRNA level and for 48 hr to detect protein level in the presence of control or HSP27 siRNA according to the manufacture instruction (RNAi group), because siRNA duplex are generally depleted in less than three days at when the interference effect of siRNA will be weak. Lysates were prepared in lysis buffer and equal amounts of protein were subjected to immunoblot analysis. Cells which were transfected with non-specific siRNA (MOCK group) or non-treated MHCC97H cells (MHCC97H group) were parallelly demonstrated.

### RT-PCR of HSP27

The procedures of RT-PCR were the same as the previous study [12]. Briefly, the total RNA of cells was extracted by Trizol (Invitrogen). cDNA synthesis was performed in 20 μl reaction system of reverse transcription including 3 μg RNA. Compound amplification of HSP27 and GAPDH acting as internal control was then carried out using equal cDNA as template. The sequence of primers is listed, HSP27: 5'-ccagagcagagtcagccagcat-3'(sense), 5'-cgaaggtgactgggatggtga-3' (antisense); GAPDH: 5'-ccatgttcgtcatgggtgtgaacca-3' (sense), 5'-gccagtagaggcagggatgatgttc-3' (antisense). The program of PCR: 95°C 30 s, 57°C 40 s, 72°C 40 s and 25 cycles were performed and PCR products were separated by 2% agarose gel electrophoresis, then scanned and analyzed by VDS imagemaster system (Pharmacia). Hsp27 primers used in this study yield an expected single band (576 bp), GAPDH primers product a 251 bp single band.

### Western blot analysis

The cell total and nuclear proteins were lysed using protein extract kit (Active Motif, USA). SDS-PAGE was performed in 12% polyacrylamide gel and proteins were transferred for 1 hr using a Bio-Rad Semi-Dry apparatus in a transfer buffer. After blocking for 1 hr with buffer (0.01 M PBS, 0.05% Tween-20 with 5% nonfat dry milk or 5% BSA), PVDF membranes were probed with anti-HSP27 (Cell Signaling Technology, USA, 1:1000), anti-phospho-HSP27 (Stressgen, USA, 1:1000), anti-activated NF-κB p65 (Kangchen Biotech, China, 1:2000), anti-IKKβ (Cell Signaling Technology, USA, 1:1000), anti-IKKα (Cell Signaling Technology, USA, 1:1000), anti-IκBα (Cell Signaling Technology, USA, 1:1000), anti-phospho-IκBα (Cell Signaling Technology, USA, 1:1000) or anti-GAPDH (Kangchen Biotech, China, 1:5000) overnight at 4°C, and incubated with HRP-conjugated secondary antibody for 1 hr at room temperature. Peroxidase activity on the PVDF membrane was visualized on X-ray film by means of the ECL Western blotting detection system.

### In vitro invasion and migration assay

In vitro invasion assay was performed using 24-well Transwell unit with polycarbonate filters (Corning Costar, Cambridge, MA). The HCC cells with RNAi treatments were placed in the upper part of the Transwell, incubated for indicated time, fixed with methanol, and stained with hematoxylin for 10 min followed briefly by eosin. Cells in the upper chamber were removed by cotton swab and the cells that invaded through the Matrigel and were located on the underside of the filter (16 fields/filter) were counted. Three invasion chambers were used per condition. The values obtained were calculated by averaging the total number of cells from three filters. Experimental procedures of in vitro migration assay are the same as the in vitro invasion assay described above except that the filter was not coated with Matrigel for the migration assay.

### Analysis of apoptotic cells by TUNEL *in situ *detection and Flow Cytometry

Cells were cultured and treated with HSP27 siRNA for 24 hr. Fix air dried cell samples with a freshly prepared solution (4% paraformaldehyde in PBS, pH 7.4) for 1 hr at 15–25°C and incubate in permeabilisation solution (0.1% Trixon X-100 in 0.1% sodium citrate) for 2 min on ice. Then add 50 μl TUNEL (Roche) reaction mixture on sample and incubate for 60 min at 37°C in a humidified atmosphere in the dark. Samples can be analyzed in a drop of PBS under a fluorescence microscope at this state.

In addition to TUNEL-staining, we attempted to confirm apoptotic rates by FACS using 5,5-6,6-tetra-chloro-1,1-3,3-tetraethylbenzimidazolyl-carbocyanine iodide (JC-1, Molecular Probes, Invitrogen Corporation, CA, USA)-staining, because the loss of mitochondrial membrane potential is an early indicator for apoptosis [13,14]. JC-1 staining solution was freshly prepared in a concentration of 10 μg/mL prior to use with DMEM medium without phenol red. Cells were cultured and treated with HSP27 siRNA for 24 hr. After washing with PBS (pH 7.4), 300 μL staining solution was added and the cells were incubated for 15 min at 37°C and 5% CO_2 _in the dark. Then washed twice in PBS, and resuspended in 300 μl PBS. The cells were then analyzed with a FACScan flow cytometer (Becton & Dickinson, Mountain View, CA, USA). JC-1 exhibits potential-dependent accumulation in mitochondria, indicated by a fluorescence emission shift from green (529 nm, FL1) to red (590 nm, FL2). In apoptotic cells, the ratio of red to green fluorescence decreases if the mitochondrial membrane depolarizes.

### cDNA microarray analysis

Total RNA was extracted from MHCC97H cells (1 × 10^6^) with different treatments (HSP27 RNAi group or MOCK group) and without treatment (MHCC97H group) using Trizol RNA extraction kit (Invitrogen). Q Series Signal Transduction in Cancer Gene Array kit (SupperArray) was used to detect the gene expression profile of signal transduction pathways. Each array membrane comprised 96 marker genes in quatraplicate; four positive controls including β-actin, GAPDH, cyclophilin A, and ribosomal protein L13α; and a negative control, bacterial plasmid pUC18. The linear normalization method was used for data analysis, based on the expression levels of four positive controls in combination with bacterial plasmid pUC18. Normalized data was log transformed and microarray spots in the t-test combined with ratio values double difference were regarded as differentially expressed genes.

### Measurement of nuclear NF-κB by ELISA

Nuclear extracts were prepared from treated and control MHCC97H cells using a nuclear extraction kit (Active Motif). Activated NF-κB in the samples was determined by oligonucleotide-based ELISA (Active Motif). Treated and control samples were normalized and incubated in 96-well microtiter wells, and the activated NFκB in the nuclear extracts attached to a consensus nucleotide sequence (5'-GGGACTTTCC-3') that was coated onto the microtiter wells. Attached NF-κB was captured by antibody to NF-κB (p65) and detected by an anti-rabbit-HRP-conjugated IgG. Color development was performed with hydrogen peroxide/TMB chromogenic substrate, and intensity of the developed color proportionately represented the quantity of NFκB in each sample. The specificity of the binding of NFκB of the samples with the nucleotide sequence was determined by comparison to the control binding, determined by adding either free consensus nucleotide or mutated nucleotide to the reaction buffer. The sample values were normalized with the total cell protein determined by protein assay kit (Pierce).

### Measurement of phospho-IκBα by ELISA

Total cell protein from each sample was normalized and was same amount which had been subjected to ELISA analysis. Phospho-IκBα ELISA kit that detects phosphorylation of Serine 32 was from Cell Signaling Technology. A mouse monoclonal antibody against IκBα was coated onto the microwells of a 96-well plate into which same amount lysates of various treated MHCC97H cells were added. Phospho-IκBα in the cell lysate was captured by the antibody, the wells were extensively washed, and a second antibody was added to detect the captured phospho(Ser32)-IκBα protein. An HRP linked anti-rabbit antibody was used to recognize the bound detection antibody, and hydrogen peroxide/TMB was added for color development. The optical density was proportional to the quantity of phospho-IκBα protein present.

### Co-immunoprecipitation Assay

Cells were rapidly trypsinized, and lysed by quick repeated freeze-thawing cycles in distilled water containing 20 mM Tris (pH 7.5), 150 mM NaCl, 1 mM EDTA, 1 mM EGTA, 1% Triton X-100, 2.5 mM Sodium pyrophosphate, 1 mM β-glycerophosphate,1 mM PMSF, 1 μg/ml Leupeptin. The cell lysate (1 mg total protein) was first precleared and next incubated with the appropriate HSP27 antibody (Cell Signaling Technology, USA, 10 μg), IKKα antibody (Cell Signaling Technology, USA, 1:100) and IKKβ antibody (Cell Signaling Technology, USA, 1:100) or goat anti-mouse/rabbit IgG1 (30 μg) (Sigma) at 4°C overnight. Protein G-Sepharose (20 μl) (Sigma) was added and the mixture was incubated for an additional 1–4 hr at 4°C. Samples were washed 3 times with buffer. The immune complexes were eluted (100 μl) with 0.1 M glycine pH 3.0 adjusted to pH 7.5 with Tris buffer and run on a SDS-PAGE, then transferred to PVDF membrane for western blot analysis.

### IKK Assay

The detection of endogenous IKK activity in HCC cells was described as the method below. Briefly, reactions were performed in 50 μl reaction volumes in 96-well polystyrene plates with final conditions as follows: 25 mM Tris-HCL (pH 7.5), 10 mM MgCL_2_, 5 mM β-glycerophosphate, 0.1 mM NaVO_3_, 2 mM DTT, 200 μM ATP, 1.5 μM biotin-IκBα peptide, and the IKK complex which was immunoprecipitated with anti- IKKα antibody(1:100). Reactions were started with IKK complex addition, incubated at room temperature for 15 min, 50 μl/well Stop Buffer (50 mM EDTA, pH 8) was added to stop the reaction. 25 μl of each reaction was transferred to a 96-well streptavidin-coated plate containing 75 μl dH_2_O/well and incubate at room temperature for 60 min. The plate was washed and incubated with 100 μl/well phosphor-IκBα mouse primary antibody (1:1000) at 37°C for 120 min, washed and HRP labeled secondary antibody in PBS/T with 1% BSA was added at room temperature for 30 min. After incubated with TMB substrate for 15 min, washed and stop solution was added. Plates were mixed well and read at 450 nm with a microtiter plate reader.

### Statistical Analysis

Data were expressed as Mean ± SE and analyzed using analysis of variance. Student's t-test was used in two-group comparisons. P < 0.05 was considered to be statistically significant.

## Results

### Up-regulated expression of HSP27 in metastatic HCC cells was positively correlated to HCC cell motility and invasion in vitro

**Figure 1:**
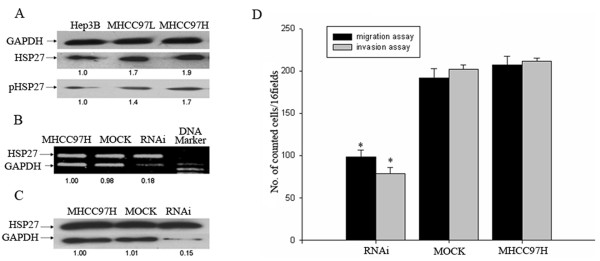
**Expression of HSP27 was distinctly inhibited after RNA interference**. Levels of HSP27 and phosphorylated HSP27 in Hep3B, MHCC97L and MHCC97H cells were up-regulated through western blot analysis of whole cell lysate using specific antibodies (**A**). PCR products of HSP27 in MHCC97H after siRNA transfection for 24 hr (**B**) and its protein expression after siRNA transfection for 48 hr (**C**) were significantly reduced in MHCC97H cells. The values on bottom of the bands represent the densitometric estimation of the relative density of the band, calculated by comparing the ratio of intensity of MHCC97L and MHCC97H cell line with that of Hep3B cell line (the ratio in Hep3B was set as baseline 1.0). In addition, MHCC97H cells transfected with siRNA for 12 hr were subjected to in vitro migration and invasion assay **(D) **for 20 hr. The results represent means of triplicates; * statistically different from control at P < 0.05.

To investigate the expression of HSP27 in Hep3B, MHCC97L and MHCC97H cells respectively with non-, high and higher metastatic potentials, we observed levels of HSP27 expression and its phosphorylation in total proteins of these HCC cells above through western blot analysis. The results showed that the level of HSP27 and basal ratio of its phosphorylation were stepwise increased from Hep3B to MHCC97L and MHCC97H cells (Fig. 1A), in parallel with metastatic potentials.

To obtain insights into whether HSP27 was directly related to HCC cells motility and invasion ability or not, knockdown of HSP27 with small interference RNA (siRNA) was performed in MHCC97H cells with higher metastatic potential. Expression of HSP27 in MHCC97H cells showed a significant decrease on the levels of mRNA (Fig. 1B) and protein (Fig. 1C) in HSP27 RNAi group, compared with MOCK group and control MHCC97H group. The results indicated expression of HSP27 in MHCC97H cells was efficiently inhibited. Then we detected cells motility and invasion in the three groups through *in vitro *migration and invasion assay. The results showed that the number of HSP27-RNAi MHCC97H cells through the filter (Migration assay: 98 ± 8; Invasion assay: 79 ± 7) was markedly less than the numbers of MOCK cells (Migration assay: 191 ± 11; Invasion assay:201 ± 5) and MHCC97H cells without any treatment (Migration assay: 207 ± 10; Invasion assay:211 ± 4) (*P *< 0.05) (Fig. 1D). Thus, inhibition of HSP27 expression suppressed significantly MHCC97H cells migration and invasion in vitro.

### Knockdown of HSP27 by siRNA induced significantly apoptosis of MHCC97H cells

**Figure 2:**
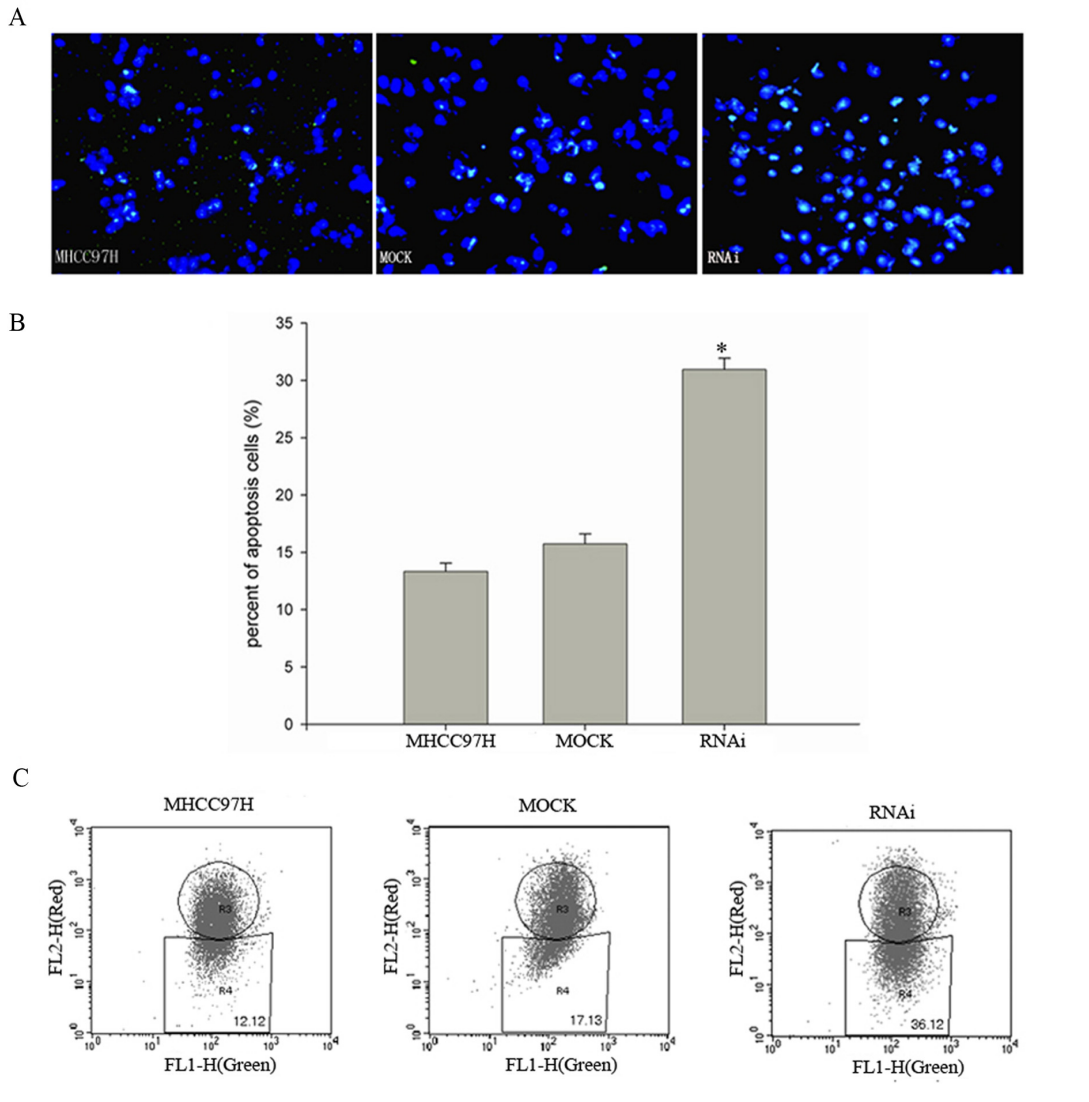
**Apoptosis ratio of MHCC97H cells increased after HSP27 RNA interference**. MHCC97H cells apoptosis were detected TUNEL technology in situ in RNAi, MOCK and MHCC97H groups (**A, B**). In addition to TUNEL-staining, apoptotic rates were confirmed by Flow Cytometry using JC-1-staining (**C**). It showed the result with respect to an early indicator for apoptosis, the loss of mitochondrial membrane potential, was consistent with the result of TUNEL detection. Data are representative of 3 separate experiments. * statistical difference from control at P < 0.05.

It is well-recognized that HSP27 is an important regulator of apoptosis pathways in cells [9]. Through TUNEL *in situ *detection, we found that apoptosis ratio of cells increased in MHCC97H cells after 24 hr treatment with siRNA against HSP27 (Fig. 2A, B), compared with MOCK group and MHCC97H cells group. In addition to TUNEL-staining, we attempted to reconfirm the apoptosis ratio of HSP27-RNAi MHCC97H with 24 hr treatment by measuring the mitochondrial membrane potential changes using JC-1-staining, because the loss of mitochondrial membrane potential is an early indicator for apoptosis[13,14]. It was shown that the percentage of apoptotic cells was increased to 36.12% in RNAi group, compared with 17.13% in MOCK group and 12.12% in control MHCC97H group (Fig. 2C). These results indicated inhibition of HSP27 expression increased HCC cells apoptosis ratio.

### Different genes from cDNA microarray analysis were prominently related to NF-κB pathway after HSP27 knockdown in MHCC97H cells

To investigate preferentially altered signal transduction pathway in MHCC97H cells after HSP27 knockdown, the Human Q Series Signal Transduction in Cancer Gene Array including marker genes with functions related to cell signal transduction pathways was used. Combined with ratio values 2-fold difference, significantly down-regulated genes including NFKB1 (NFκB), IL2, NFKBIA (IκBα), LTA, PECAM1 passed statistic t-test. The results were reconfirmed by real-time PCR with different and specific primer pairs [see Additional file 1]. Through Pathway Miner software analysis, these down-regulated genes indicated the preferential change of NF-κB pathway after inhibition of HSP27 expression in MHCC97H cells.

### HSP27 was involved in NF-κB pathway activation in HCC cells

**Figure 3:**
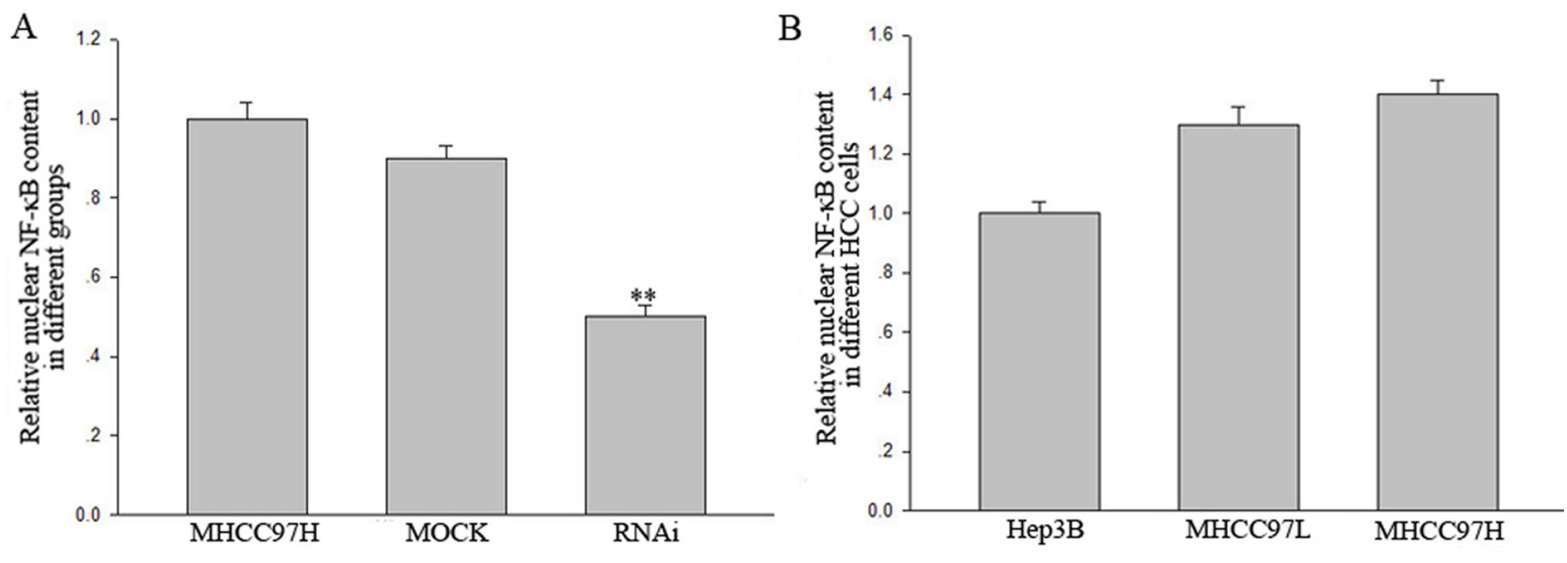
**HSP27 was involved in activation of NF-kB pathway in HCC cell lines**. Nuclear NF-κB concentration was analyzed by ELISA in HSP27 RNAi, MOCK and MHCC97H cells (A) and in Hep3B, MHCC97L and MHCC97H cells (B). The values represent the relative intensity measured at 450 nm of these different treated cell lysates. The results represent means of triplicates. ** statistical difference from control at P < 0.01.

Microarray analysis results promoted us to elucidate a role of HSP27 on activation of NF-κB pathway in HCC cells. Firstly, after HSP27 knockdown, it was found that level of activated NF-κB in nuclei decreased about 0.5-fold in MHCC97H cells (Fig. 3A). Then, concentration of nuclear NF-κB was detected in Hep3B, MHCC97L and MHCC97H cells. It showed there was an increased tendency of nuclear NF-κB concentration from Hep3B to MHCC97H cells (Fig. 3B), which was identical to consecutively up-regulated expression pattern of HSP27 in the three cell lines (Fig. 1A). These results suggested that HSP27 may have an effect on NF-κB activation in metastatic HCC cells.

**Figure 4:**
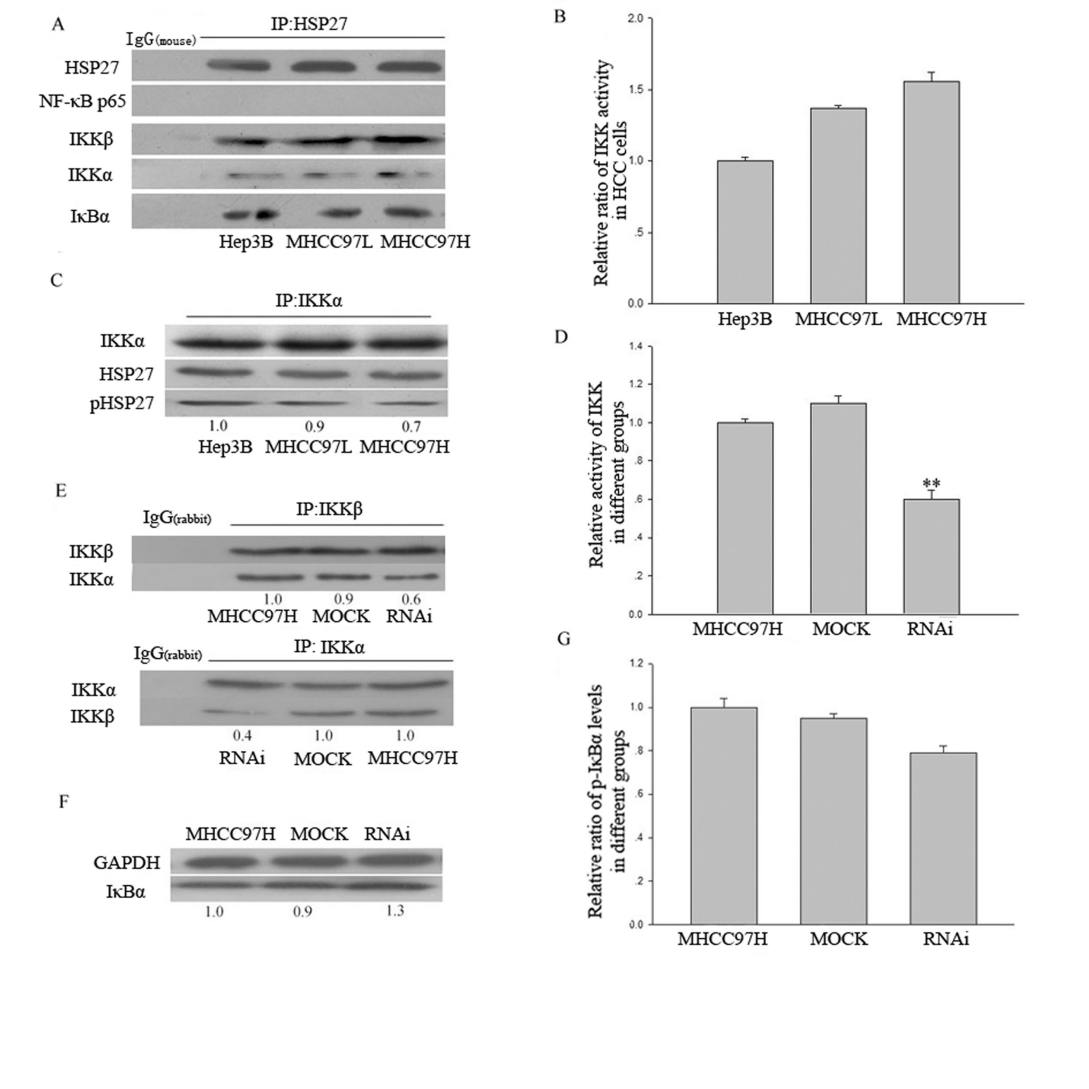
**HSP27 interacts with key molecular (IKKβ, IKKα, IκBα) of NF-kB pathway in HCC cell lines**. The precipitated IKKβ, IKKα, IκBα with anti-HSP27 antibody or normal mouse IgG1 were detected by western blot analysis in Hep3B, MHCC97L and MHCC97H cells, the HSP27 contents in the precipitates were used as equal loading amount control (**A**). Endogenous IKK activity in Hep3B, MHCC97L and MHCC97H cells (**B**) and in RNAi, MOCK and MHCC97H cells (**D**) was detected through IKK assay. The absorbed value of reaction solution from MHCC97H cells or Hep3B cells served as a percent control. The precipitated HSP27 and phosphorylated HSP27 with IKK complex by using anti-IKKα antibody were detected in Hep3B, MHCC97L and MHCC97H cells, the IKKα contents in the precipitates were used as equal loading amount control (**C**). Moreover, the precipitated IKKβ with anti-IKKα antibody or precipitated IKKα with anti-IKKβ antibody and normal rabbit IgG1 in RNAi, MOCK and MHCC97H cells were demonstrated by western blot analysis and the association between IKKα and IKKβ was reduced. The IKKα or IKKβ contents in the precipitates were used as equal loading amount control (**E**). In addition, total IκBα (**F**) and phosphorylated IκBα (**G**) were also analyzed by western blot analysis or ELISA in RNAi, MOCK and MHCC97H cells. The values on bottom of the bands represent the densitometric estimation of the relative density of the band. Data are representative of 3 separate experiments; * statistical difference from control at P < 0.05.

To further confirmed this idea, immunoprecipitates from Hep3B, MHCC97L and MHCC97H cells using HSP27 antibody were analyzed by SDS-PAGE, followed by western blot analysis with activated NF-κB p65, IKKα, IKKβ and IκBα-specific antibodies. As shown in Fig. 4A, IKKα, IKKβ and IκBα were detected out in the immunoprecipitates, but not activated NF-κB p65. Further IKK assay analysis showed that IKK activity was highest in MHCC97H cells, followed by MHCC97L cells and then Hep3B cells (Fig. 4B), which was consistent with the elevated tendency of NF-κB activation in the three HCC cell lines. In addition, phosphorylation of HSP27 in immunoprecipitates from Hep3B, MHCC97L, MHCC97H cells was determined by western blot analysis with IKKα antibody, it was found that phosphorylated HSP27 which was associated with IKK complex reduced from Hep3B, MHCC97L to MHCC97H cells (Fig. 4C).

In addition, IKK activity reduced significantly (Fig. 4D) and the association between IKKβ and IKKα decreased (Fig. 4E) after HSP27 knockdown in MHCC97H cells, compared with MOCK group. Moreover, it was shown that protein level of total IκBα increased 1.3-fold (Fig. 4F), but its phosphorylated level tended to slightly reduced (0.8-fold) after HSP27 knockdown in MHCC97H cells, compared with MOCK group cells (Fig. 4G).

## Discussion

Although HSP27 is induced transiently after periods of cell stress, it is often constitutively over-expressed in tumor cells [9,15]. In addition, increased HSP27 is responsible for the increase in experimental endothelial cell metastasis and further associated with metastatic disease in breast and ovarian cancers [16,17]. Our previous studies and this study showed that up-regulated expression of HSP27 was related to malignant potentials of HCC cells [10,12,18], it suggested a role of HSP27 in HCC development and progression.

It is hypothesized that tumor cells with elevated levels of heat-shock proteins have a selective pro-survival advantage that contributes to the process of tumorigenesis and enhancement of malignant potentials. Moreover, the precondition of gaining such advantages requires persistent activation of proliferation signal or blockage of apoptosis pathway [7,19]. It is also well-known that HSP27 is able to block apoptosis at different stages because of its interaction with a number of partners implicated in the apoptotic pathways [20,21]. Furthermore, the capacity of HSP27 to interact with one or another partner seems to be determined by the oligomerization/phosphorylation status which is two activation forms of HSP27 [22-24]. Through flow cytometry and TUNEL label analysis, we found that apoptotic ratio of metastatic HCC cells increased significantly after inhibition of HSP27 expression. The data suggested an important role of up-regulated HSP27 in inhibiting HCC cells apoptosis.

To elucidate the effect of up-regulated HSP27 on HCC cells apoptosis, through a signal transduction cDNA microarray analysis, we found that these significantly different genes were prominently related to NF-κB signal transduction pathway in HCC cells. It seems likely that regulation of HSP27 on NF-κB pathway activation is involved in metastatic HCC cells apoptosis.

It is reported that IκB kinase/NF-κB (IKK/NF-κB) signaling pathway plays critical roles in a variety of physiological and pathological processes. During the process, the control of IκBα phosphorylation in response to all other NF-κB activating stimuli rests on the shoulders of the IκB kinase (IKK) complex [25]. Moreover, the association of NF-κB pathway activation with tumor promotion, progression, and metastasis was well documented [26]. In diverse cell types, NF-κB signaling pathway has been shown to have a critical role in regulating the apoptotic program. Whether NF-κB promotes or inhibits apoptosis appears to depend on the specific cell type and the type of inducer [27]. In this research, we observed that activated NF-κB in nuclei increased gradually from Hep3B, MHCC97L to MHCC97H cells. It is accordance with some studies in HCC [28]. More intriguingly, we have found that the level of nuclear activated NF-κB reduced after HSP27 knockdown, indicating depletion of HSP27 inhibited NF-κB pathway activation in HCC cells. It was reported that, as a component of IKK complex, Hsp90/Cdc37 was related to regulation of IKK activity, indicating heat shock protein could be involved in regulation of NF-κB signal pathway activation [29]. Therefore, we next examined effects of HSP27 on key molecules of NF-κB pathway.

We demonstrated that HSP27 was associated with IKKα, IKKβ and IκBα in HCC cells. The result indicated that effects of HSP27 on NF-κB pathway activation may be from its interaction with IKKα, IKKβ and IκBα. It was also found in this study that basal level of phosphorylated HSP27 and IKK activity increased consecutively from Hep3B to MHCC97L, MHCC97H cells, which was consistent with activated NF-κB in nuclei. It suggested NF-κB pathway activation may be IKK-dependent in our metastatic HCC cell lines. In addition, IKK complex in HCC cells was not only associated with phosphorylated HSP27, but with non-phosphorylated HSP27, and it also showed that phosphorylated HSP27 associated with IKK complex reduced from Hep3B, MHCC97L to MHCC97H cells. Therefore, it was possible that phosphorylation of HSP27 may have a negative effect on activation of IKK complex through interacting with some intercellular kinases [30], whereas non-phosphorylated HSP27 may maintain IKK signalosome specific conformation or assist the IKK resolubilization in HCC cells, which can be implied by suppression of endogenous IKK complex activity in HSP27-knockdown HCC cells. Alternatively, it has been reported that HSP90 could regulate IKK receptiveness to post-translational modification or even the activity of enzymes that carry out such modifications [31]. Whether HSP27 plays such a role in HCC cells needs further study.

In addition, our findings also showed, after HSP27 depletion by siRNA, the level of total IκBα mildly increased but its phosphorylated ratio showed a reduced tendency in HCC cells. It suggested that HSP27 may play dual roles in IκBα degradation, not only favors a proteasome complex for IκBα degradation through direct association with IκBα, but also enhances indirectly IκBα phosphorylation through IKK complex.

## Conclusion

In summary, the results reported in the present study suggested at some extent the contribution of HSP27 to metastatic HCC cells apoptosis, which involves more than one single mechanism. Particularly relevant in this regard is the regulation of HSP27 on key molecules of NF-κB signaling pathway. This study provides a new insight for analyzing the role of HSP27 in HCC cells resistance against apoptosis.

## Abbreviations

HCC: Hepatocellular carcinoma; HSP: heat shock protein; RNAi: RNA interference; siRNA: small interference RNA; JC-1: 5,5-6,6-tetra-chloro-1,1-3,3-tetraethylb enzimidazolyl-carbocyanine iodide; RT-PCR: transcription polymerase chain reaction; TUNEL: terminal deoxinucleotidyl transferase dUTP nick end-labelling; ELISA: enzyme-linked immunosorbent assay.

## Competing interests

The authors declare that they have no competing interests.

## Authors' contributions

GK was responsible for most of the experimental work and drafted the manuscript. KX and LY participated in the design of this study. SL and CJ assisted in the statistical analysis. GL and GD participated in culturing various metastatic HCC cells and immunoassay detection. LY supervised this study, and involved in revising it critically for important intellectual content. All authors read and approved the final manuscript.

## Pre-publication history

The pre-publication history for this paper can be accessed here:

http://www.biomedcentral.com/1471-2407/9/100/prepub

## Supplementary Material

Additional file 1 **Validation of microarray results with specific primers of selected genes for real time PCR.** The data represented real time PCR programs details of selected genes with specific primers and the results were consistent with the microarray results. Click here for file
