# Supplementary figures and images for: Regulation of HSP27 on NF-κB pathway activation may be involved in metastatic hepatocellular carcinoma cells apoptosis
Source: BMC Cancer. 2009 Mar 31;9:100. doi: 10.1186/1471-2407-9-100 (PMC2681475; doi:10.1186/1471-2407-9-100)

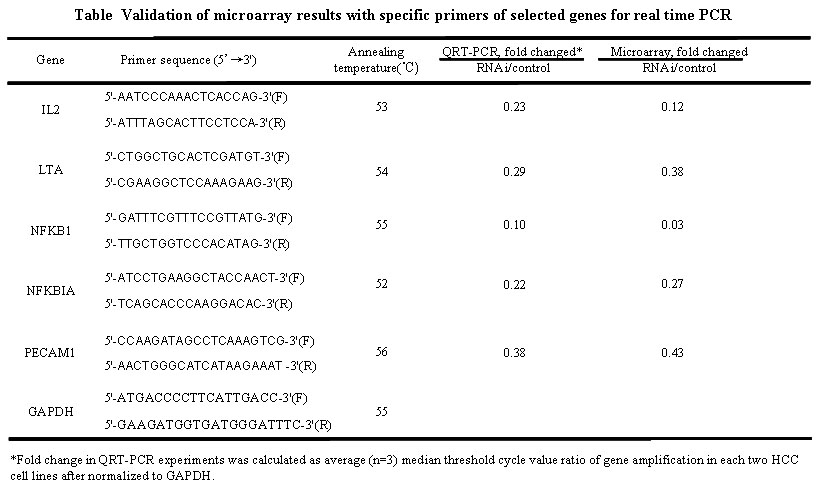

Supplement: Additional file 1 — Validation of microarray results with specific primers of selected genes for real time PCR. The data represented real time PCR programs details of selected genes with specific primers and the results were consistent with the microarray results. [file 1471-2407-9-100-S1.doc]
